# Human CD4^+^ Memory T Cells Can Become CD4^+^IL-9^+^ T Cells

**DOI:** 10.1371/journal.pone.0008706

**Published:** 2010-01-14

**Authors:** Prabhakar Putheti, Amit Awasthi, Joyce Popoola, Wenda Gao, Terry B. Strom

**Affiliations:** 1 Department of Medicine, Transplant Institute, Beth Israel Deaconess Medical Center, Harvard Medical School, Boston, Massachusetts, United States of America; 2 Center for Neurologic Diseases, Brigham and Women's Hospital, Harvard Medical School, Boston, Massachusetts, United States of America; New York University, United States of America

## Abstract

**Background:**

IL-9 is a growth factor for T- and mast-cells that is secreted by human Th2 cells. We recently reported that IL-4+TGF-β directs mouse CD4^+^CD25^−^CD62L^+^ T cells to commit to inflammatory IL-9 producing CD4^+^ T cells.

**Methodology/Principal Findings:**

Here we show that human inducible regulatory T cells (iTregs) also express IL-9. IL-4+TGF-β induced higher levels of IL-9 expression in plate bound-anti-CD3 mAb (pbCD3)/soluble-anti-CD28 mAb (sCD28) activated human resting memory CD4^+^CD25^−^CD45RO^+^ T cells as compared to naïve CD4^+^CD25^−^CD45RA^+^ T cells. In addition, as compared to pbCD3/sCD28 plus TGF-β stimulation, IL-4+TGF-β stimulated memory CD4^+^CD25^−^CD45RO^+^ T cells expressed reduced FOXP3 protein. As analyzed by pre-amplification boosted single-cell real-time PCR, human CD4^+^IL-9^+^ T cells expressed GATA3 and RORC, but not IL-10, IL-13, IFNγ or IL-17A/F. Attempts to optimize IL-9 production by pbCD3/sCD28 and IL-4+TGF-β stimulated resting memory CD4^+^ T cells demonstrated that the addition of IL-1β, IL-12, and IL-21 further enhance IL-9 production.

**Conclusions/Significance:**

Taken together these data show both the differences and similarities between mouse and human CD4^+^IL9^+^ T cells and reaffirm the powerful influence of inflammatory cytokines to shape the response of activated CD4^+^ T cells to antigen.

## Introduction

Cytokines present within the milieu in which human naïve CD4^+^ T cells are activated via stimulation of the TCR complex and co-stimulatory molecules dictate whether these cells commit to Th1, Th2, Th17 or regulatory (Treg) T cell phenotype [1]–[4]. The commitment of mouse and human CD4^+^ T cells to such distinct phenotypes is directed through expression of lineage specific transcription factors, e.g., Tbet for Th1, GATA3 for Th2, and RORγt for Th17 cells [5]–[7]. While IL-4 and TGF-β respectively direct activated mouse and human naïve CD4^+^ T cells to commit to the Th2 or inducible Treg (iTreg) phenotypes, the presence of IL-4+TGF-β directs mouse CD4^+^CD25^−^CD62L^+^ T cells or CD4^+^CD25^−^CD44^low^ T cells to commit to supposedly a novel inflammatory IL-9 producing CD4^+^ T cell subset (“Th9”) [8]–[10]. Mouse studies did not examine the effects of IL-4+TGF-β in fostering commitment of naïve CD4^+^CD25^−^CD62L^high^CD44^low^ *versus* resting memory CD4^+^CD25^−^CD62L^low^CD44^high^ T cells to CD4^+^IL-9^+^ T cells [8], [10]. IL-9 was initially cloned as a T-cell growth factor and IL-9 receptor shares the common γ-chain with other IL-2 family members IL-2, -4, -7, -15, and -21 [11], [12]. It is yet to be examined if IL-4+TGF-β induce a “Th9” like subset in humans.

We hypothesized that IL-4+TGF-β in the setting of plate bound-anti-CD3 mAb (pbCD3)/soluble-anti-CD28 mAb (sCD28) stimulation fosters commitment of human naïve and resting memory CD4^+^CD25^−^ T cells to express IL-9. Our analysis which includes a successful effort to optimize IL-9 expression or frequency of CD4^+^IL-9^+^ T cells and their molecular phenotype reveals both similarities and distinctions between human and murine IL-9^+^ T cells. In addition, we have determined that resting and memory CD4^+^ T cells are not equal in their frequency to express IL-9. While examining the requirements to optimize generation of human CD4^+^IL-9^+^ T cells, we found that IL-1β, IL-12, or IL-21, that foster commitment to Th1 or Th17 subsets, enhance IL-4+TGF-β induced CD4^+^IL-9^+^ T cell generation in presence of pbCD3/sCD28 stimulation.

## Materials and Methods

### Ethics Statement

This study was conducted according to the principles expressed in the Declaration of Helsinki. The study was approved by the Institutional Review Board of Beth Israel Deaconess Medical Center. All adult healthy volunteers provided written informed consent for the collection of samples at Children's Hospital Blood Donor Center, Boston, MA, and for subsequent analysis.

### Media, Monoclonal Antibodies, and Cytokines

RPMI 1640 (Gibco, Paisley, Scotland) with L-glutamine was supplemented with 1% non-essential amino acids, 10% FCS, 50 U/ml penicillin and 50 µg/ml streptomycin (all from Gibco BRL, Maryland, USA) and used as culture medium. The following anti-human mAbs were used: Anti-CD3 (UCHT1) and anti-CD28 (CD28.2); Peridinin chlorophyll protein (PerCP, red fluorescence) cy5.5 labeled anti-CD4 (BD biosciences); phycoerythrin (PE, orange fluorescence)-labeled anti-IL-10, anti-IFNγ, anti-IL-17, anti-IL-9 (Biolegend); Alexa-488- or fluorescence isothiocyanate (FITC, green fluorescence)-labelled anti-FOXP3 and anti-Annexin-V (Biolegend). Irrelevant isotype-matched mouse mAbs were purchased from Biolegend: FITC-IgG1, PE-IgG2a, PE-cy5-IgG2a, and PerCP-IgG1. Recombinant IL-1β, IL-2, IL-4, IL-6, IL-12, IL-21, TGF-β, and neutralizing anti-IFNγ, -IL-2, and -IL-4 mAb were purchased from R&D.

### Preparation of Mononuclear Cells

Leukofilters were obtained from fifteen adult healthy volunteers. Leukocytes were obtained by back-washing cells from leukofilters with Dulbecco's phosphate buffered saline (PBS) (Gibco). CD4^+^ T cells were collected from leukocytes using *Rosette-sep Human CD4^+^ T cell isolation kit* (Stemcell technologies) and density gradient centrifugation on Lymphoprep (Nycomed, Oslo, Norway). The cells from the interphase were collected and washed three times with PBS, and trace erythrocytes were removed by hypotonic lysis. Cell viability as measured by Trypan blue exclusion and the purity of CD4^+^ T cell isolation as analyzed by flow cytometry always exceeded 95%.

### Isolation of CD4^+^CD25^−^ T Cells

Isolated CD4^+^ T cells were then stained with biotinylated-anti-CD25 mAb, and CD25^+^ T cells were depleted using *Easy-sep biotin selection kit* (Stemcell technologies). The purity of CD4^+^CD25^−^ T cell isolation was always >99%, as analyzed by flow-cytometry. To isolate naïve CD4^+^ T cells, CD4^+^CD25^−^ T cells were stained with biotinylated–anti-CD45RO mAb and then CD45RO^+^ T cells were depleted using *Easy-sep biotin selection kits*; similarly to isolate resting memory CD4^+^ T cells, CD4^+^CD25^−^ T cells were stained with biotinylated–anti-CD45RA mAb and CD45RA^+^ T cells were depleted using *Easy-sep biotin selection kits*.

### In Vitro Generation of Polarized Human Th1, Th2, Th17, and iTreg Cells

In cell culture assays, 1.0×10^6^/ml CD4^+^CD25^−^ T cells, CD4^+^CD25^−^CD45RA^+^ T cells, or CD4^+^CD25^−^CD45RO^+^ T cells were activated with 5 µg/ml pbCD3 and 1 µg/ml sCD28 in culture media at 37°C/5%CO2. To promote CD4^+^ T cell differentiation and proliferation, 1.0×10^6^/ml CD4^+^CD25^−^ T cells were cultured for 4 days in the presence of rIL-2 (1.5ng/ml) and either rIL-12 (8ng/ml) plus anti-IL-4 mAb (10µg/ml) for Th1-polarizing conditions or rIL-4 (20ng/ml) plus anti-IFNγ mAb (10µg/ml) for Th2-polarizing conditions, or rhIL-6 (20ng/ml), rhIL-1β (10ng/ml), anti-IL-4 plus anti-IFNγ mAb for Th17-polarizing conditions, or rTGF-β (1ng/ml) for iTreg-polarizing condition. The resulting Th1, Th2, Th17 cells were tested for expression of IFNγ, IL-4, IL-17 cytokines by flow cytometry and expression of Tbet, GATA3, RORC, and FOXP3 transcripts by quantitative real-time PCR (qt-RT-PCR). In addition, 1.0×10^6^/ml CD4^+^CD25^−^ T cells, naive CD4^+^CD25^−^CD45RA^+^ T cells, or memory CD4^+^CD25^−^CD45RO^+^ T cells were cultured in presence of pbCD3/sCD28 activation and rIL-4 (20ng/ml) plus rTGF-β (1ng/ml). In some experiments, memory CD4^+^CD25^−^CD45RO^+^ T cells were activated with pbCD3/sCD28 and IL-4+TGF-β for 96hrs in presence or absence of rhIL-1β (10ng/ml), rhIL-2 (1.5ng/ml), rhIL-6 (20ng/ml), rIL-12 (8ng/ml), or rIL-21 (50ng/ml). At 96hrs, supernatants of memory CD4^+^CD25^−^CD45RO^+^ T cell cultures were collected for quantitation of IFNγ, IL-2, IL-5, IL-9, IL-10, IL-13, and IL-17 by ELISA. A portion of cells in these cultures were treated with RNA*later* for quantitation of IL-9 transcripts by qt-RT-PCR, and the remainder of this cell population was resuspended in FCS supplemented with 10% DMSO (Sigma) and these cells were frozen at −80°C until flow-cytometric analysis of the phenotype.

### Measurement of Cytokines by ELISA and Intracellular Ccytokine Staining

Culture supernatants were assessed for cytokine production by ELISA (Aushon BioSystems). To detect expression of surface or intracellular proteins, cells were stained with commercial mAb as per manufacturer's instructions (eBioscience). Cells were analyzed by FACScan or LSRII.

### RNA Extraction and cDNA Synthesis

Frozen cells were thawed and homogenized using 25-gauge syringe. Total RNA was extracted using Invitrogen's Purelink micro to midi kit (Carlsbad, CA) and the RNA concentration was measured on Nanodrop ND 1000 spectrophotometer (Wilmington, DE). 1ug of total RNA was converted into cDNA using Taqman Reverse Transcription kit (Applied Biosystems, NJ)

### Quantitative TaqMan Real Time PCR

The ABI PRISM 7900HT Sequence Detection System was used for qt-RT-PCR analysis. All primer-probe (P&P) sets were custom designed, except IL-9 and GAPDH (Applied Biosystems). Custom designed P&P sets were validated by serially diluting cDNA isolated from cells expressing the target and verifying the slope, and by sequencing the amplicon. Taqman Fast PCR Master Mix was purchased from Qiagen (Valencia, CA). Amplification was carried out in a total volume of 25 µl for 40 cycles of 3 seconds at 95°C, 30 seconds at 60°C. Initial denaturation was performed for 3 min at 95°C. Target gene expression was normalized by 18s rRNA (house keeping gene (HKG)) expression.

### Single Cell Real-Time PCR

Single cell PCR was performed as per manufacturer's instructions (Pre-Amp Cells-to-Ct kit, Applied Biosystems). Briefly, cultured cells were stained with Annexin-V for live/dead cell gating and live Annexin-V^−^ single cells were sorted by FACS-Aria into 96 well V bottom plate containing 8µl lysis solution (containing DNAse I). One to 32 cells per well were collected in triplicates for generating a standard curve for HKG, and cycle threshold (Ct) value of HKG for a single cell was determined. Accordingly, test samples with Ct value of HKG that fell in range of a single cell were chosen for analyzing expression of target genes. Reverse transcription and pre-amplification were done as per manufacturer's instructions. Qt-RT-PCR was performed as mentioned above. T-bet, GATA3, RORC, IFNγ, IL-9, IL-10, IL-13, IL-17A, and IL-17F transcripts were quantitated on single CD4^+^ T cells. We normalized the target gene expression by GAPDH.

### Statistical Analysis

Multiple groups were compared by the non-parametric Kruskal-Wallis ANOVA; in case *p<0.05* was obtained, differences between pairs of groups were further compared by the Mann-Whitney U-test or Dunn's test. When data from only three independent experiments (n = 3) were analyzed, unpaired *t*-test with Welch's correction for unequal variances was used. In any case, differences were considered statistically significant if *p<0.05*.

## Results and Discussion

### IL-4+TGF-β Induces In-Vitro Generation of pbCD3/sCD28 Activated Human CD4^+^IL-9^+^ T Cells

**Figure 1:**
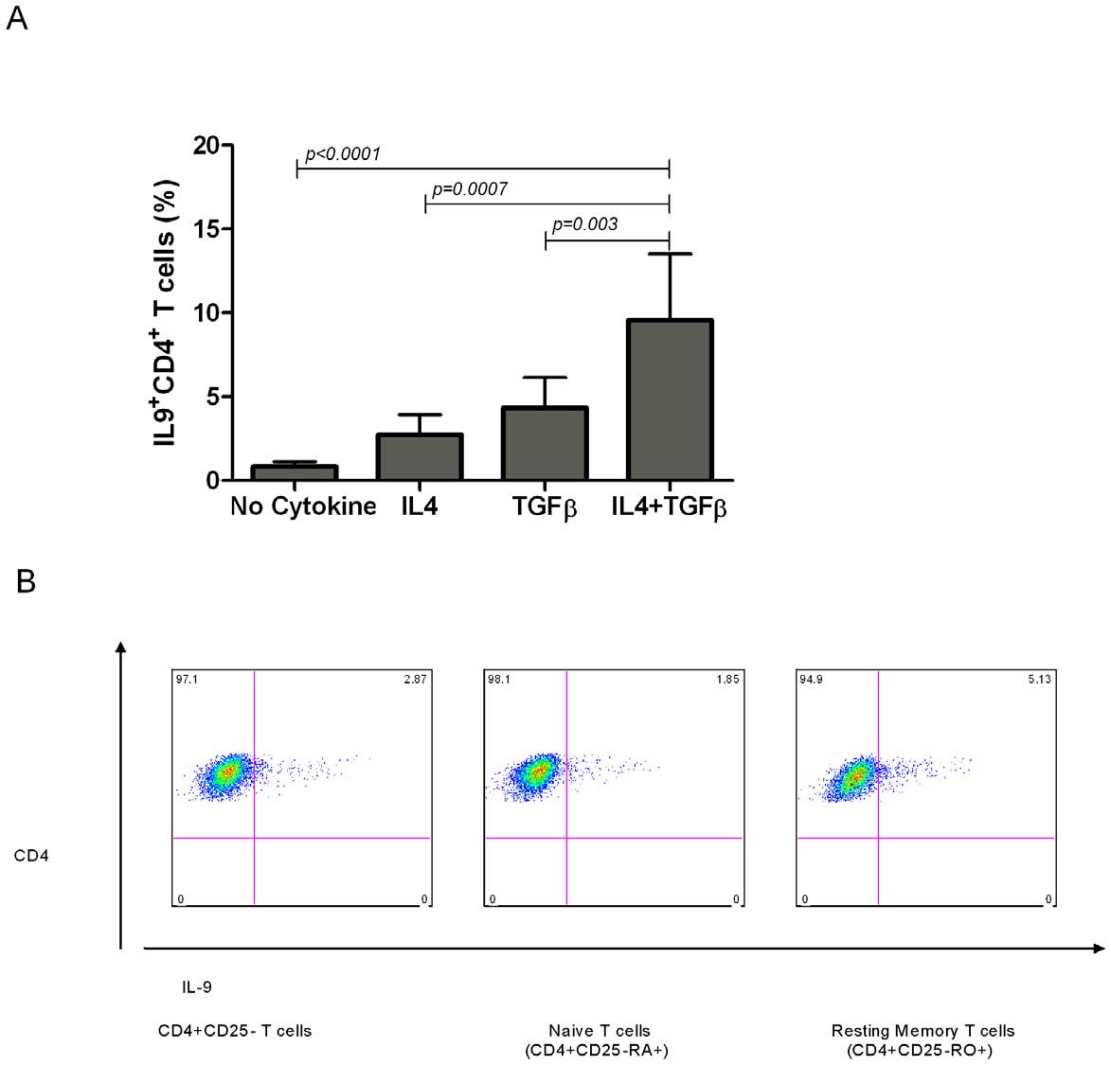
IL-4+TGF-β in presence of pbCD3/sCD28 activation induce generation of CD4^+^IL-9^+^ T cells. **A)** CD4^+^CD25^−^ T cells (1.0×10^6^/ml in 24 well plates) were activated with plate bound-anti-CD3 mAb (pbCD3)/soluble-anti-CD28 mAb (sCD28) in presence or absence of IL-4 or TGF-β or IL-4+TGF-β for 96hrs and analyzed by flow cytometry for IL-9 expression. IL-4+TGF-β in combination induced significantly higher percentage of CD4^+^ T cells positive for IL-9, as compared to IL-4, TGF-β, or neither (*n = 14*). Data is expressed as the mean±SD. **B)** CD4^+^CD25^−^ T cells, CD4^+^CD25^−^CD45RA^+^ T cells (naïve T cells), CD4^+^CD25^−^CD45RO^+^ T cells (resting memory T cells) (2.0×10^5^/ml in 96 well plates) were activated with pbCD3/sCD28 in presence of IL-4+TGF-β in a 96 well plate for 96hrs. Cells were surface stained for CD4 PerCP-Cy5.5 and intracellular stained for IL-9 PE. IL-4+TGF-β in combination induced IL-9 expression by both naïve and memory T cells, but memory T cells expressed high levels of IL-9. Data are representative of six independent experiments (six different donors).

IL-9 produced by mouse Tregs mediate recruiting mast cells to the tissue niche and regulates effector T cells during graft rejection [13]. Neutralization of IL-9 in vivo in this setting abrogates Treg cell function and promotes graft rejection [13]. On the other hand, there is compelling data from the mouse study suggesting the potentiality of “Th9” cells in inducing colitis and peripheral neuritis [8]. In humans, in-vitro IL-4+IL-10, in the presence of anti-CD3 and B7-1/FcγRII-transfected fibroblasts, induces naïve or memory CD4^+^ T cells to produce IL-9 [14]. However, if this induction of IL-9 production was dependent or independent of TGF-β was not examined. Moreover, an assessment of the expression of lineage specification factors and other cytokines by human CD4^+^IL-9^+^ T cells has not been undertaken. Hence, there is a need for systematic analysis of human CD4^+^IL-9^+^ T cells differentiation. To address this, we activated freshly isolated CD4^+^CD25^−^ T cells for 96hrs with pbCD3/sCD28 in presence or absence of IL-4 or TGF-β or IL-4+TGF-β. As shown in figure 1A, IL-4- or TGF-β-alone induced generation of 2% or 4% of CD4^+^IL-9^+^ T cells, whereas no IL-9 was expressed in the absence of IL-4 or TGF-β. Interestingly, IL-4+TGF-β induced expression of IL-9 in up to 10% of pbCD3/sCD28 activated CD4^+^ T cells.

Human resting CD4^+^ T cells (CD4^+^CD25^−^ T cells) are comprised of naïve CD4^+^CD25^−^CD45RA^+^ T cells and resting memory CD4^+^CD25^−^CD45RO^+^ T cells. We separated CD4^+^CD25^−^CD45RA^+^ naïve T cells from CD4^+^CD25^−^CD45RO^+^ resting memory T cells and activated with IL-4+TGF-β and pbCD3/sCD28 for 96hrs. We found that IL-4+TGF-β in combination induced IL-9 expression in both naïve and memory CD4^+^ T cells, but higher percentage of memory CD4^+^ T cells expressed IL-9.

**Figure 2:**
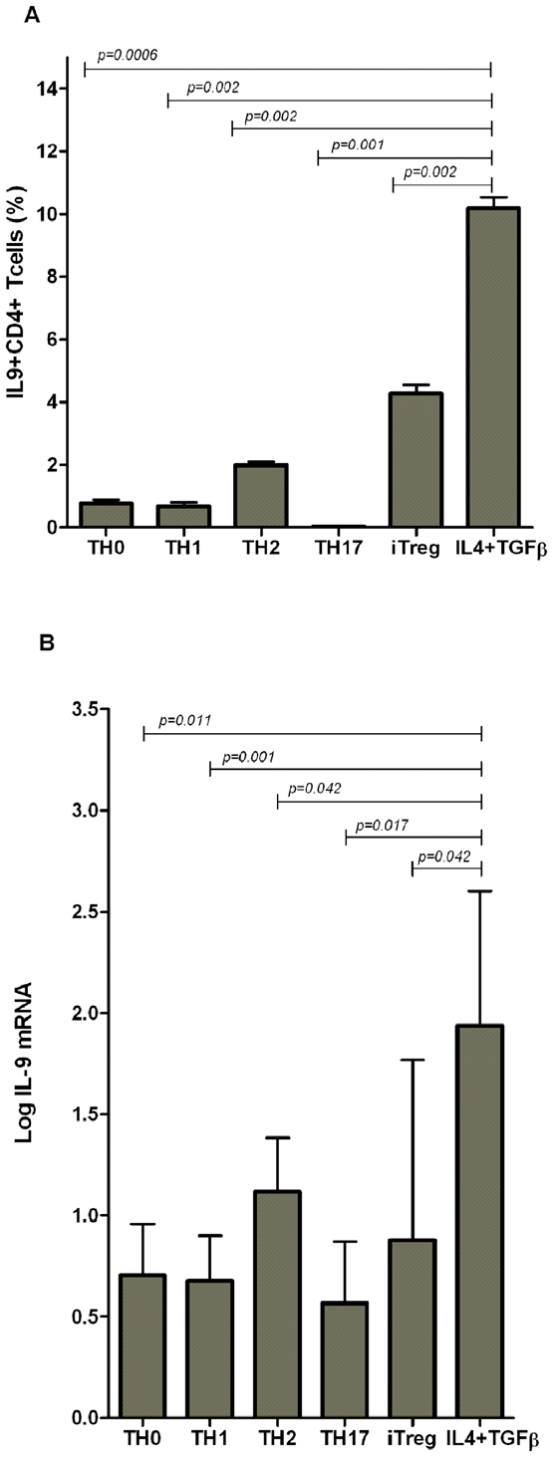
CD4^+^CD25^−^ T cells activated with IL-4+TGF-β express more IL-9 than Th1, Th2, Th17, or iTregs. CD4^+^CD25^−^ T cells (1.0×10^6^/ml in 24 well plates) were activated with pbCD3/sCD28 in presence or absence of IL-4+TGF-β or Th1-, Th2-, Th17-, iTreg-polarizing condition for 96hrs. Cells were harvested, gated on CD4^+^ T cells, and were analyzed for IL-9^+^ cells by flow cytometry or were used to quantitate IL-9 transcripts by real-time PCR. Data is expressed as the mean±SD. (A) 2% of Th2 cells, 4% of iTregs, or 10% of cells treated with IL-4+TGF-β in combination were IL-9^+^, whereas Th1-, Th17-, or Th0-cells had negligible number of IL-9^+^ cells (*n = 7*); (B) Log-transformed ratios of IL-9 mRNA copies to 18S rRNA are shown. Cells treated with IL-4+TGF-β in combination had significantly higher levels of IL-9 mRNA as compared to polarized Th1-, Th2-, Th17-, iTreg-, or Th0-cells (*n* = 9).

IL-9 is produced by mouse and human Th2 cells [15], and by mouse nTregs [13]. We then examined for IL-9 production by human CD4^+^CD25^−^ T cells cultured in-vitro in Th1-, Th2-, and Th17-cell polarizing conditions. Robust IL-9 expression was detected in IL-4+TGF-β treated CD4^+^CD25^−^ T cells at both mRNA and protein levels as compared to Th0- (*p = 0.0006* and *p = 0.01*), Th1- (*p = 0.002* and *p = 0.001*), Th2- (*p = 0.002* and *p = 0.04*), Th17- (*p = 0.001* and *p = 0.01*) cells, and iTregs (*p = 0.002* and *p = 0.04*) (Fig 2). To our knowledge, this is the first report showing IL-9 expression by human iTregs.

### Cytokine Profiles of IL-4+TGF-β Treated CD4^+^CD25^−^CD45RO^+^ T Cells

**Figure 3:**
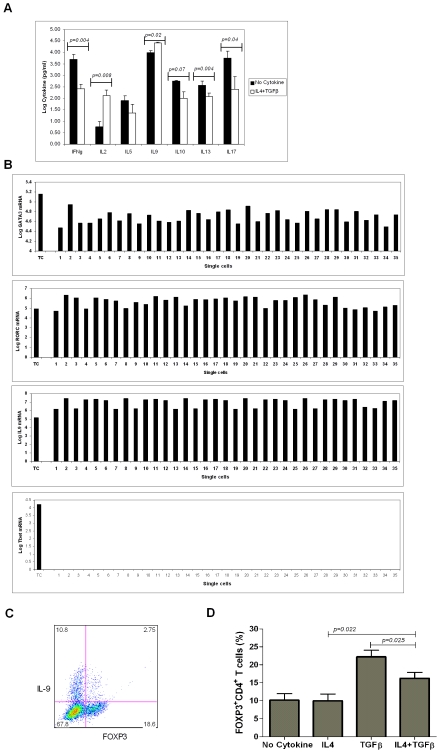
Cytokine and transcription factor profile of memory CD4^+^CD25^−^CD45RO^+^ T cells activated with IL-4+TGF-β. **A)** Log-transformed quantities of cytokines (pg/ml) are shown. CD4^+^CD25^−^CD45RO^+^ T cells (1.0×10^6^/ml in 24 well plates) were activated with pbCD3/sCD28 in presence or absence of IL-4+TGF-β. Supernatants were collected at 96hrs post activation and IFNγ, IL-2, IL-5, IL-9, IL-10, IL-13, and IL-17 were quantified by ELISA. IL-4+TGF-β treated CD4^+^ T cells produced significantly high IL-2 and IL-9, but significantly low IFNγ, IL-13, and IL-17, as compared to CD4^+^CD25^−^CD45RO^+^ T cells not treated with IL-4+TGF-β (*n = 3*). Data is expressed as the mean±SD. **B)** Log-transformed ratios of mRNA copies to GAPDH mRNA copies for GATA3, RORC, IL-9, and Tbet are shown. CD4^+^CD25^−^CD45RO^+^ T cells (1.0×10^6^/ml in 24 well plates) were activated with pbCD3/sCD28 in presence of IL-4+TGF-β. Cells were harvested and single cell sorted. IL-9 transcripts were quantified by qt-RT-PCR. 10,000 cells comprising of total cell population (TC) was also taken and the gene expression was averaged for single cell for reference. Cells positive for IL-9 transcripts were further quantitated for GATA3, RORC, and Tbet (*n* = 3). As IL-9 is expressed in only 10% of all CD4^+^ T cells activated with pbCD3/sCD28 in presence of IL-4+TGF-β, average IL-9 mRNA copies of TC are always lower than that of a single IL-9^+^ cell. CD4^+^IL-9^+^ T cells expressed GATA3 and RORC, but not Tbet. **C)** CD4^+^CD25^−^CD45RO^+^ T cells (1.0×10^6^/ml in 24 well plates) were activated with pbCD3/sCD28 in presence of IL-4+TGF-β. Cells were surface stained for CD4 and intracellular stained for IL-9 and FOXP3. Cells were gated for CD4 and then IL-9^+^ or/and FOXP3^+^ cells were analyzed. 25% of CD4^+^IL-9^+^ T cells were also FOXP3^+^. Data are representative of seven independent experiments. **D)** CD4^+^CD25^−^CD45RO^+^ T cells (1.0×10^6^/ml in 24 well plates) were activated with pbCD3/sCD28 in presence or absence of IL-4 or TGF-β or IL-4 plus TGF-β for 96hrs and analyzed by flow cytometry for FOXP3 expression. IL-4 significantly inhibited TGF-β induced FOXP3 expression (*n = 3*). Data is expressed as the mean±SD.

Next, we examined the cytokine profile of these IL-9 producing resting memory CD4^+^CD25^−^CD45RO^+^ T cells 96hrs after in-vitro stimulation with pbCD3/sCD28 in presence of IL-4+TGF-β. In this setting, activated memory CD4^+^CD25^−^CD45RO^+^ T cells secreted low levels of IFNγ (*p = 0.004*), IL-13 (*p = 0.004*), IL-17 (*p = 0.004*), IL-5 and IL-10 (not statistically significant) in comparison to control cultures lacking IL-4+TGF-β, as analyzed by ELISA (Fig 3A). Note that in the presence of IL-4+TGF-β and pbCD3/sCD28 stimulation, memory CD4^+^CD25^−^CD45RO^+^ T cells secreted higher levels of IL-2 as compared to control cultures lacking IL-4+TGF-β (*p = 0.008*). This suggests a possible role of IL-2 in maintaining viability of CD4^+^IL-9^+^T cells generated from resting memory CD4^+^CD25^−^CD45RO^+^ T cells. As IL-9 is expressed by human Th2 cells and mouse “Th9” cells produced IL-10, we hypothesized that TGF-β would synergize with IL-4 in inducing production of all the Th2 cytokines. We found that TGF-β synergized with IL-4 in elevating IL-9 production, but inhibited IL-4 mediated IL-5, IL-10, and IL-13 production.

### CD4^+^IL-9^+^ T Cells Express GATA3 and RORC

We probed human CD4^+^IL-9^+^ T cells generated from IL-4+TGF-β stimulated resting memory CD4^+^CD25^−^CD45RO^+^ T cells for expression of the Tbet, GATA3, and RORC lineage specification transcription factors by pre-amplification boosted single cell qt-RT-PCR. These CD4^+^IL-9^+^ T cells are GATA3^+^, RORC^+^, Tbet^−^ (Fig 3B), IFNγ^−^, IL-10^−^, IL-13^−^, IL-17A^−^, and IL-17F^−^ (data not shown). In keeping with reports showing that RORγt expression is not limited to Th17 cells [16], we note expression of RORC, mRNA for RORγt, in in-vitro generated human CD4^+^IL-9^+^ T cells, although these cells do not express IL-17A or IL-17F expression.

IL-4 alone induces GATA3^+^ Th2 differentiation. We show that TGF-β doesn't blunt IL-4 mediated induction of GATA-3 (Fig 3B), yet TGF-β inhibits IL-4 mediated IL-13 expression (Fig 3A). This suggests that TGF-β acts downstream of GATA-3 to prevent IL-4 mediated IL-13 production. TGF-β, a cytokine present in many inflammatory states, in combination with the pro-inflammatory IL-6 cytokine induces generation of inflammatory Th17 cells [7], [17]. We now demonstrate that TGF-β and IL-4, an anti-inflammatory cytokine, induce expression of human CD4^+^IL-9^+^ T cells and inhibit expression of Th2 cytokines.

As deduced by intracellular staining, approximately 25% of CD4^+^IL-9^+^ T cells are FOXP3^+^ (Fig 3C). In humans, FOXP3 is transiently expressed by many in-vitro activated T cells [18] and iTregs can be generated from both naïve CD4^+^CD25^−^CD45RA^+^ T cells and resting memory CD4^+^CD25^−^CD45RO^+^ T cells [18], [19]. Among the cell donors, the proportion of memory CD4^+^CD25^−^CD45RO^+^ T cells expressing FOXP3 upon 96 hrs of pbCD3/sCD28 stimulation in presence of TGF-β was ∼20–40%. Although, IL-4 did not induce FOXP3 expression among memory CD4^+^CD25^−^CD45RO^+^ T cells in presence of pbCD3/sCD28 stimulation, IL-4 inhibited TGF-β induced FOXP3 expression in this setting (*p = 0.025*) (Fig 3D) in line with mouse report [8]. This suggests that IL-4 and TGF-β inhibit each other's effects, and skew the cells to CD4^+^IL-9^+^ T cells.

### Influence of IL-1β, IL-2, IL-6, IL-12, IL-21 on CD4^+^IL-9^+^T Cell Generation

**Figure 4:**
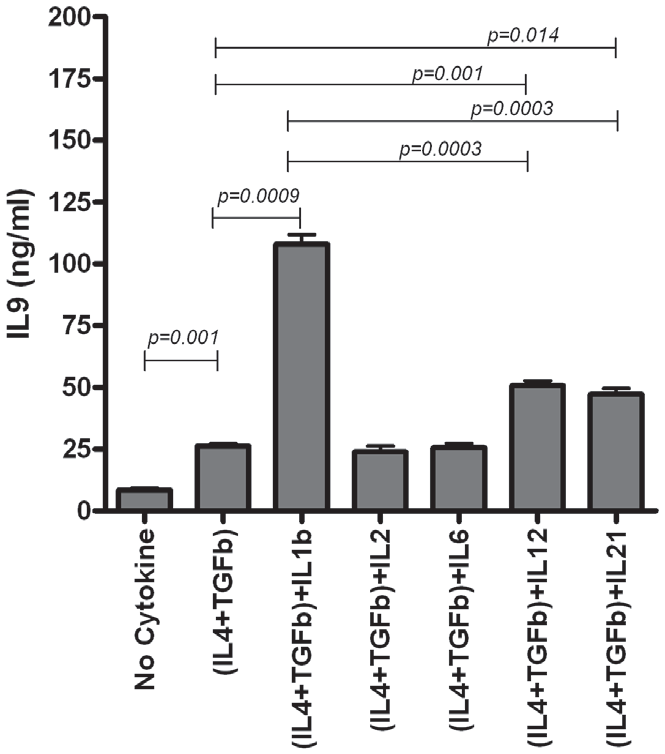
IL-1β amplifies IL-4+TGF-β induced IL-9 production by memory CD4^+^CD25^−^CD45RO^+^ T cells. Resting memory CD4^+^CD25^−^CD45RO^+^ T cells (2.0×10^5^/ml in 96 well plates) activated with pbCD3/sCD28 alone or with IL-4+TGF-β, in the presence or absence of IL-1β, IL-2, IL-6, IL-12, and IL-21 for 96hrs and supernatants were collected. Influence of IL-1β, IL-2, IL-6, IL-12, and IL-21 on IL-9 production of IL-4+TGF-β treated CD4^+^CD25^−^CD45RO^+^ T cells was examined. IL-1β, IL-12 or IL-21 significantly elevated IL-4+TGF-β induced IL-9 production, but IL-1β had significantly higher influence compared to IL-12 or IL-21 (*n = 3*). Data is expressed as the mean±SD.

As IL-9 is a pro-inflammatory cytokine, we then hypothesized that cytokines IL-12, IL-1β, IL-6, and IL21, that induce inflammatory Th1 or Th17 cell polarization, would enhance IL-4+TGF-β mediated generation of CD4^+^IL-9^+^ T cells in presence of pbCD3/sCD28 activation. IL-6 did not influence IL-9 secretion. IL-1α and IL-1β induce IL-9 production by activated murine-CD4^+^ T cells or -mast cells [15], [20]. IL-1β also stimulates IL-9 production by human eosinophils [21]. Strikingly, we found that IL-1β, in the presence of IL-4+TGF-β, induced four fold more IL-9 secretion by IL-4+TGF-β+pbCD3/sCD28-activated resting memory CD4^+^ T cells as compared to control cultures lacking IL-1β (*p = 0.0009*) (Fig 4). As macrophages are the major producers of IL-1β, this suggests their plausible role in stimulation of human CD4^+^IL-9^+^ T cells. We examined if IL-1β alone or in combination with either IL-4 or TGF-β is sufficient to induce IL-9 expression. As analyzed by flow cytometry, IL-4 or TGF-β induced generation of 2% or 4% of CD4^+^IL-9^+^ T cells did not alter upon adding IL-1β to the cultures, and no IL-9 was expressed in the presence of IL-1β alone (data not shown). Albeit to a lesser effect, IL-12 or IL-21 also amplified IL-9 secretion by IL-4+TGF-β+pbCD3/sCD28-activated memory CD4^+^CD25^−^CD45RO^+^ T cells (*p = 0.001 or p = 0.014*). As IL-2 was robustly secreted by IL-4+TGF-β treated resting memory CD4^+^ T cells (Fig 3A), it is not totally unexpected that addition of IL-2 to cultures of resting memory CD4^+^CD25^−^CD45RO^+^ T cells activated with pbCD3/sCD28 and IL-4+TGF-β did not have any significant effect on IL-9 secretion (Fig 4). Addition of neutralizing anti-IL-2 mAb to cultures had lead to CD4^+^ T cell death (data not shown). This data suggests IL-2 is required to maintain viability of IL-9 producers and IL-2 may not directly trigger IL-9 expression. Over all, anti-inflammatory cytokines, IL-4 and TGF-β, induce IL-9 production, and pro-inflammatory cytokines, IL-1β, IL-12, and IL-21, enhance IL-9 production.

In conclusion, IL-4+TGF-β, a combination that has proved effective in generating “Th9” subset in mouse systems, in pbCD3/sCD28 activated human CD4^+^CD25^−^CD45RO^+^ T cells induced high levels of IL-9 expression. Th2 cells and iTreg were the only other human CD4^+^ T cell subsets that expressed IL-9. This study doesn't conclude that dual influence of IL-4 and TGF-β in presence of TCR stimulation and co-stimulation induces human “Th9” cell subset, but like in mouse, does induce CD4^+^IL-9^+^ T cells. Further knowledge of specific-transcription factor and/or –cell surface marker is required to state these cells as human novel “Th9” cells. With more than one subset of CD4^+^ T cells producing IL-9, existence of “Th9” cells as a new subset is debatable. But, so far both mouse and human results suggest that IL-9 expression is not devoid of IL-4 or TGF-β stimulation. In-so-far, these CD4^+^IL-9^+^ T cells generated from memory CD4^+^ T cells are different from mouse CD4^+^IL-9^+^ T cells generated from naïve CD4^+^ T cells in the following aspects: 1) human CD4^+^IL-9^+^ T cells express GATA3 and RORC; 2) some human CD4^+^IL-9^+^ T cells express FOXP3 and; 3) human CD4^+^IL-9^+^ T cells do not express IL-10. On the other hand, this study shows certain similarities between mouse and human systems, where like in mouse, human CD4^+^IL-9^+^ T cells do not express Tbet, IFNγ, IL-5, and IL-13 and; IL-4 inhibits TGF-β induced FOXP3 expression. We did not address if human CD4^+^IL-9^+^ T cells are functionally effector cells like in mouse. Attempts to optimize IL-9 production by pbCD3/sCD28 and IL-4+TGF-β stimulated resting memory CD4+ T cells demonstrated that the addition of enhances IL-9 production. These data revive the potent influence of pro-inflammatory cytokines IL-1β, IL-12, and IL-21 to modulate the response of antigen activated CD4^+^ T cells.
